# Efficacy of Quality and Quantity media-cultured mononuclear cells for promoting peripheral nerve regeneration in mouse model

**DOI:** 10.1371/journal.pone.0321457

**Published:** 2025-04-16

**Authors:** Nutthawut Akaranuchat, Nuttapol Chruewkamlow, Chutipon Sathan-ard, Phakawan Phutthakunphithak, Sompol Tapechum, Chanean Ruangsetakit, Nuttawut Sermsathanasawadi

**Affiliations:** 1 Division of Plastic and Reconstructive Surgery, Department of Surgery, Faculty of Medicine Siriraj Hospital, Mahidol University, Bangkok, Thailand; 2 Siriraj Center for Regenerative Medicine, Research Department, Faculty of Medicine Siriraj Hospital, Mahidol University, Bangkok, Thailand; 3 Department of Physiology, Faculty of Medicine Siriraj Hospital, Mahidol University, Bangkok, Thailand; 4 Division of Vascular Surgery, Department of Surgery, Faculty of Medicine Siriraj Hospital, Mahidol University, Bangkok, Thailand; China Medical University, TAIWAN

## Abstract

This study aimed to assess the efficacy of Quality and Quantity media-cultured mononuclear cells (QQ-MNCs) for promoting nerve regeneration in a mouse sciatic nerve transection model. Human peripheral blood mononuclear cells (PB-MNCs) and QQ-MNCs derived from healthy volunteers were used/compared. The left sciatic nerve was surgically transected in 27 mice. After complete nerve transection was confirmed, end-to-end direct epineurial nerve repair was performed using 9–0 nylon. Fibrin glue was applied to the tissue around the injury site to limit diffusion of the study treatment followed by application of 0.5 ml phosphate buffered saline (PBS) or PB-MNCs (2x10^6^ cells) or QQ-MNCs (2x10^6^ cells) to the injury site. The skin was then closed using 6–0 nylon. Histomorphology, immunohistochemistry, electrophysiologic examination, and functional assessment were evaluated at 12-weeks followed by euthanasia and subsequent harvesting of the left sciatic nerves and the left and right gastrocnemius muscles for examination. QQ-MNCs mice exhibited significant improvement in all histomorphologic parameters (axon fiber diameter, myelin thickness, percentage of nerve density) and immunohistochemistry assays (S100, SOX10, GFAP, neurofilament, IL-1β, VEGF, anti-HNA, TNF-α, vWF) compared to PBS mice (all *p* < 0.05). QQ-MNCs mice also had a significantly higher Basso Mouse Scale score compared to PBS mice (*p* = 0.018). The percentage of nerve density adjacent to the injury site was significantly higher in QQ-MNCs mice than in PB-MNCs mice (*p* = 0.049). IL-1β expression was significantly lower in QQ-MNCs mice than in PB-MNCs mice (*p* = 0.01). QQ-MNCs mice demonstrated significantly better functional and histomorphologic outcomes of nerve regeneration compared to PB-MNCs mice and PBS mice.

## Introduction

Peripheral nerve injury is a common problem in medical practice with a reported incidence of 13–23 events per 100,000 persons per year [1–3]. Peripheral nerve injury is most commonly caused by sharp injury, high-impact injury, or as a consequence of medical conditions, such as adverse event after tumor extirpation or chronic disease (e.g., diabetes mellitus). Patients with peripheral nerve injury will commonly experience abnormal sensation, paresthesia, and/or loss of function of the affected organ or limb [4,5]. The current established methods for treating/managing peripheral nerve injury include primary neurorrhaphy, nerve autografts, and tuberization techniques using a nonabsorbable or absorbable tube. However – after treatment, many patients still have permanent weakness, paresthesia, and/or malfunction of the affected organ or limb. It is, therefore, imperative that we identify and implement new strategies for promoting nerve regeneration to shorten the recovery time, to improve the results of treatment, and to improve patient quality of life.

Stem cells are capable of self-renewal and of differentiating into any cell type [6]. These characteristics suggest stem cells as a potential therapy for enhancing the outcomes of nerve regeneration and for augmenting the effects of conventional treatments. Moreover, stem cells are a known source of Schwann cells, which produce growth factors and cytokines, and that can upregulate and downregulate immunomodulatory response [7,8].

In 2009, Kijima, *et al*. reported that CD133^ +^ cells could significantly enhance nerve regeneration and decrease recovery time after injury compared to mononuclear cells (MNCs) or phosphate buffered saline (PBS) [4]. In 2017, Kamei, *et al*. reported that endothelial progenitor cells (EPCs) and MNCs could enhance myelination, increase axon growth, and facilitate angiogenesis [5]. Other researchers also reported good outcomes of MNCs for nerve regeneration [9–11].

Quality and Quantity mononuclear cells (QQ-MNCs), which are MNCs that are cultured in Quality and Quantity (QQ) culture media, were reported to have both increased numbers of progenitor cells and enhanced anti-inflammatory effects [12]. QQ-MNCs were also shown to improve vasculogenesis and angiogenesis [13]. However and to date, no study has reported the investigation of QQ-MNCs for promoting peripheral nerve regeneration. Accordingly, the aim of this study was to investigate the efficacy of QQ-MNCs for promoting peripheral nerve regeneration compared to human peripheral blood MNCs (PB-MNCs) or PBS (control) in a mouse sciatic nerve transection model.

## Materials and methods

### Study design and ethical approval

This prospective experimental animal study was conducted at the Faculty of Medicine Siriraj Hospital, Mahidol University, Bangkok, Thailand during the February 2023 to March 2024 study period. Siriraj Hospital is Thailand’s largest national tertiary referral center. The protocol for this study was approved by the Siriraj Institutional Review Board (certificate of approval number Si 591/2022), and fully complied with all of the principles set forth in the 1964 Declaration of Helsinki and all of its subsequent amendments.

### Healthy human volunteers

Four healthy human volunteers (age range: 20–40 years/ 3 males, 1 female) were prospectively recruited at the Faculty of Medicine Siriraj Hospital, Mahidol University, Bangkok, Thailand during February 2023 to June 2023. Written informed consent was obtained from all volunteers prior to their participation in this study.

### Cell culture

Twenty milliliters (ml) of heparinized peripheral blood were collected by venous puncture of the superficial vein at the forearm. PB-MNCs were isolated by density gradient centrifugation using Lymphocyte Separation Solution (Sigma-Aldrich Corporation, St. Louis, MO, USA). PB-MNCs at a concentration of 2 × 10^6^ cells/2 ml were cultured in QQ culture media for 7 days. QQ culture media is composed of Stem Line II Solution (Sigma-Aldrich) and five recombinant human proteins including stem cell factor, thrombopoietin, Flt-3 ligand, vascular endothelial growth factor, and IL-6 (all PeproTech, Cranbury, NJ, USA) [14,15]. In the PB-MNCs group, PB-MNCs were cultured in 10% fetal bovine serum (FBS) and Roswell Park Memorial Institute (RPMI)-1640 Medium [14,15].

### Phenotypic analysis

After 7 days of culturing, cells were harvested and washed twice with 2% FBS and 0.02% sodium azide (NaN_3_) PBS. Ten microliters (μl) of FcR blocking reagent (Miltenyi Biotec, Bergisch Gladbach, Germany) was added, after which the cells were suspended in a buffer consisting of 2 millimoles per liter (mmol/L) of ethylenediaminetetraacetic acid (EDTA), 0.2% bovine serum albumin (BSA), and PBS followed by incubation at 4 degrees Celsius (°C) for 30 minutes. The cells were then stained with a combination of monoclonal antibodies that are the markers of progenitor cells (CD34 + CD133 + cells), M2 macrophages (CD206 + cells), and inactivated T regulatory cells (CD4 + CD25 + CD127 + cells).

### Phenotypic analysis of progenitor cells and M2 macrophages

Phenotypic analysis of progenitor cells and M2 macrophages was performed using the first panel. Cells were incubated with 5 μl of each monoclonal antibody (mAb) at 4°C for 30 minutes. The mAbs used were CD34-FITC (#343504; BioLegend, San Diego, CA, USA), CD11c-PE (#371504; BioLegend), CD133-APC (#372806; BioLegend), CD3-PE-Cy7 (300420; BioLegend), CD206-APC-Cy7 (#321119; BioLegend), and CD11b-PerCP Cy5.5 (#101228; BioLegend). The cells were washed with 2% FBS and 0.02% NaN_3_ PBS two times and then fixed with 1% paraformaldehyde (Sigma-Aldrich) in PBS [14,16].

### Phenotypic analysis of inactivated T regulatory cells

Phenotypic analysis of inactivated regulatory T (CD4 + CD25 + CD127+) cells was conducted using the second panel. Cells were incubated with 5 μl of each mAb at 4°C for 30 minutes. The antibodies used were CD25-PE (#302606; Bio- Legend), CD127-APC (#351316; BioLegend), CD3-Pe-Cy7 (#300420; BioLegend), and CD4-APC-Cy7 (#300518; BioLegend) [14,16].

Cell assessment was performed using a BD LSR Fortessa Flow Cytometer^TM^ (BD Biosciences, San Jose, CA, USA). All experiments were conducted in triplicate. The percentages of CD34 + CD133 + cells, CD206 + cells, and CD4 + CD25 + CD127 + cells in PB-MNCs cultured in QQ culture media were compared with PB-MNCs cultured in standard culture media.

### Endothelial progenitor cell (EPC) colony formation assay (EPC-CFA)

After being subjected to the *ex vivo* serum-free expansion culture system (QQc), the cultured cells were further subjected to an endothelial progenitor cell (EPC) colony formation assay (CFA) using MethoCult SF H4236 medium (STEMCELL Technologies, Vancouver, Canada). This semisolid culture medium was supplemented with various factors to support colony formation, including basic fibroblast growth factor (bFGF), recombinant human vascular endothelial growth factor (rhVEGF), recombinant human stem cell factor (rhSCF), recombinant human insulin-like growth factor-1 (rhIGF-1), recombinant human interleukin-3 (rhIL-3), and recombinant human epidermal growth factor (rhEGF) (all PeproTech, Cranbury, NJ, USA). The medium also contained 30% FBS, heparin, and an antibiotic cocktail.

The EPC-CFA working medium was mixed with 30% FBS-Iscove’s Modified Dulbecco’s Medium (IMDM) and then seeded into 35 mm Primaria culture dishes (density of 2 × 10^5^ cells per dish) (Corning, Inc., Corning, NY, USA). Seeding was performed using blunt-end needles and 1 mL syringes to facilitate cell adhesion.

On the 14th day, the number of adherent colonies per dish were counted. A gridded scoring dish and a phase-contrast light microscope were used to calculate endothelial progenitor cell colony-forming cells (EPC-CFCs). A colony was defined as the presence of 50 or more cells. The experiments were repeated 3 times and the mean values were recorded to compare colony numbers between PB-MNCs and QQ-MNCs.

### Mouse sciatic nerve transection model

Female BALB/c nude mice aged 10 weeks (weight range: 19–22 grams) were used in this study. The study mice were obtained from Nomura Siam International Co., Ltd. (Bangkok, Thailand). Prior to the surgical procedure, study mice were maintained in a 25°C environment with a 12/12 light/dark cycle, and both water and food (murine chow diet) were available ad libitum. Ethical approval for animal experimentation was obtained and all relevant animal use and care guidelines were carefully followed and applied.

The left sciatic nerve was surgically transected in 27 mice. There were 9 mice in the QQ-MNC group (study), 9 mice in the PB-MNC group (study), and 9 mice in the PBS group (control). Induction and maintenance of anesthesia during the procedure was performed by administration of ketamine (80 mg/kg) and xylazine (10 mg/kg) via the intraperitoneal route. The surgical area at the groin of the left side hindlimb was prepared after which the skin over the groin crease was incised. The sciatic nerve was then identified under a 20x surgical microscope. Once identified, the sciatic nerve was cut at 5 mm above trifurcation. Electrophysiologic examination, including nerve conduction velocity (NCV) and threshold of gastrocnemius muscle stimulation, was used to confirm complete sciatic nerve transection. After complete nerve transection was confirmed, end-to-end direct epineurial nerve repair was performed using 9–0 nylon suture in 2 stitches. Fibrin glue was then applied to the tissue around the injury site to help contain and limit the diffusion of the subsequent study treatment infusion. The study treatment infusion [0.5 ml PBS or PB-MNCs (2x10^6^ cells) or QQ-MNCs (2x10^6^ cells)] was then applied to the nerve injury site and its immediate surroundings. The skin incision was then closed using 6–0 nylon sutures. The study mice received ketoprofen 10 mg/kg, which is a non-steroidal anti-inflammatory drug (NSAID), to control postoperative pain.

The study mice were maintained in the same conditions during the 12-week postoperative period as they were prior to undergoing nerve transection and repair. At the 12-week time point, the study mice underwent functional and electrophysiologic assessment. The mice were then euthanized after which both the left and right sciatic nerves and gastrocnemius muscles were harvested for examination (a surgical microscope was used to identify the suture site so that area could be harvested for histological examination). The left sciatic nerve specimen from the experimental site was divided into 2 pieces – the proximal nerve and the distal nerve. The proximal nerve specimen originated at the nerve repair site to a distance of 5 mm, and the distal nerve specimen originated at 5 mm to 10 mm of nerve distally to the site of the neurorrhaphy. The total duration of time expended for experimentation in this study was 1 year, and we used a total of 30 mice (3 mice died during the experiment, 2 died during sciatic nerve surgery, and 1 died at 6-weeks after surgery due to an unknown cause of death). During the study period, study mice were maintained in a 25°C environment with a 12/12 light/dark cycle, and both water and food (murine chow diet) were available ad libitum. During the postoperative period, all study mice received ketoprofen 10 mg/kg, which is a non-steroidal anti-inflammatory drug (NSAID), to control postoperative pain. The postoperative condition of study mice was checked every day. Food, water, and habitat were changed at least once per week. Health and behavior monitoring of the animals was performed every day by our research team and a veterinarian. After completion of the experiment at the 12-week time point, all mice were sacrificed by cervical dislocation method.

### Functional assessment

Functional assessment was performed using the Basso Mouse Scale (BMS) and Sciatic Nerve Functional Index (SFI) at 12 weeks after sciatic nerve transection and repair [4,6]. A clear acrylic walking track (45 cm long x 8 cm wide x 15 cm high) was used to assess mouse behavior, activity, gait, and footprints [17]. All mouse movements were recorded in video files using a Sony A7 camera (Sony Corporation, Tokyo, Japan) that was positioned under the clear acrylic walking track to capture mouse movements from below. The analysis of BMS and SFI was performed by an expert in animal model research [18–24] who was blinded to the mouse study group assignment.

### Electrophysiologic examination

**Fig 1:**
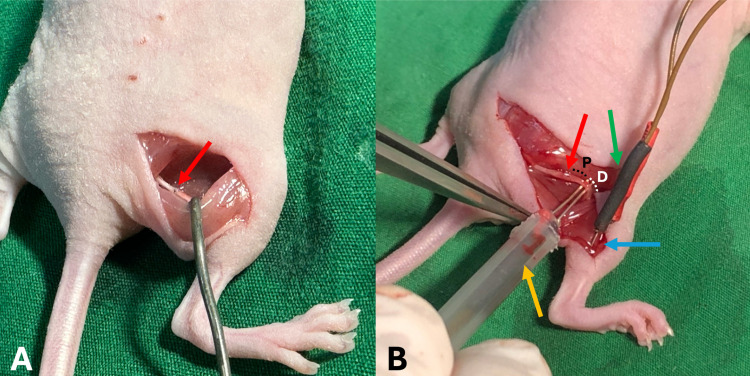
Electrophysiological evaluation of the transected sciatic nerve. (A) Sciatic nerve at 12 weeks after primary neurorrhaphy (the red arrow indicates the location of sciatic nerve transection and repair). (B) Electrophysiologic examination (the red arrow indicates the sciatic nerve, the yellow arrow indicates the electrical stimulation probe, the green arrow indicates the recording electrodes penetrating the gastrocnemius muscle, and the blue arrow indicates the gastrocnemius muscle) (“P” indicated area of proximal nerve specimen, and “D” indicated area of distal nerve specimen).

After functional assessment, the study mice were anesthetized followed by sciatic nerve threshold testing and nerve conduction velocity (NCV) to the gastrocnemius muscle. Briefly, the sciatic nerve was identified as described previously. The needle-recording electrodes were inserted into the gastrocnemius muscle for compound muscle action potential (CMAP) recording. The proximal and distal recording electrodes were located just proximal and distal to the sciatic nerve anastomotic site, respectively. A PowerLab recording unit with Chart software (ADInstruments, Castle Hill, Australia) was used for data acquisition [25,26] (Fig 1). The sciatic nerve threshold and maximal stimuli were identified during the increment of stimulus voltage stimulations. A stimulus intensity of 75% of the maximum stimulus was used for the NCV study. The latencies of both proximal and distal stimulation were identified offline. The NCV was calculated by the dividing the distance between the proximal and distal stimulating electrodes by the outcomes from the delta latency between proximal and distal stimulations.

### Histomorphologic and immunohistochemistry assessment

Sciatic nerve specimens were embedded and underwent a frozen section technique (5 µm thickness per cut). Histomorphologic staining to assess nerve and Schwann cell morphology was performed using hematoxylin & eosin (H&E) stain (to represent the morphology and architecture) and modified Gomori trichrome (mGT) stain (to identify the myelinated fiber). The immunohistochemistry examinations were performed using anti-S100 (Anti-S100 Alpha Antibody [EPR5251], Abcam Limited, Cambridge, United Kingdom) to detect Schwann cell density; anti-SOX10 (Anti-SOX10 Antibody [EPR4007–104], Abcam) for identification of neural crest cells, the precursors of Schwann cells; anti-GFAP (Anti-GFAP Antibody [EPR1034Y], Abcam) to demonstrate immature Schwann cells and non-myelinating Schwann cells; anti-neurofilament (Anti-160 kD Neurofilament Medium Antibody [EPR23510–76], Abcam) to assess the number and density of axons; anti-VEGF (Anti-VEGFA Antibody [EP1176Y], Abcam) for the detection of endothelial cells and capillary vessel formation; anti-vWF (Anti-von Willebrand Factor Antibody [sc-53465], Santa Cruz Biotechnology, Dallas TX, USA) to assess angiogenesis; and, anti-IL-1β (Anti-IL-1 Beta Antibody [EPR23851–127], Abcam) and anti-TNF-α (Anti-TNF Alpha Antibody [52B83], Abcam) to assess inflammation at and adjacent to the nerve transection and repair site. Anti-HNA (Anti-Human Nuclear Antigen Antibody [235-1], Abcam) staining was used to evaluate the viability and resignation of human stem cells in our mouse model. The primary antibody was counterstained with Alexa Fluor 488 (Goat Anti-Rabbit IgG H&L, Abcam) and Alexa Fluor 647 (Goat Anti-Mouse IgG H&L, Abcam) [27]. For the analysis of the percentage of nerve density, the axon fiber diameter, the myelin thickness, and the muscle fiber diameter, the histological images were taken from five selected areas of each slide under 40X. Then, the images were converted by Image J software. The percentage of nerve density was measured by recording the integrated optical density (IOD) of the nerve fiber in the selected area by adjusting the gradient to measure the area containing axons. The percentage of area containing axons in the image was calculated and then averaged for each slide. The axon fiber diameter, the myelin thickness, and the muscle fiber diameter were then measured and averaged for each slide. For the analysis of immunohistochemistry staining, the process was performed by recording the IOD of each parameter. The values will represent the amount of immunoreactivity. All measurements and comparisons were performed using ImageJ software (National Institutes of Health, Bethesda, Maryland, USA) to evaluate the outcomes [28–30].

The wet-weight of gastrocnemius muscle specimens from both the left and right legs was recorded immediately after resection from the euthanized mice. The percentage of wet-weight muscle retention was calculated by subtracting the weight of the normal side from the delta weight of the normal and experimental side, and then dividing that value by the weight of the normal side.

All gastrocnemius muscle specimens were embedded and underwent the same frozen section method as the nerve specimens. Histomorphologic staining was performed, including H&E (to represent the morphology and architecture) and mGT (to differentiate the muscle nuclei from muscle myofibrils). Immunohistochemistry examination was performed using anti-VEGF, anti-vWF, anti-IL-1β, and anti-TNF-α to assess angiogenesis and inflammation. The outcomes were evaluated and measured using ImageJ software.

### Sample size calculation and statistical analysis

To assess the efficacy of QQ-MNCs with a 5% alpha error and a 10% allowable error, our sample size calculation indicated a minimum sample size of 8–9 mice per group, so 27 mice were purchased for use in this study (9 mice per group) [4].

All statistical analyses were performed using SPSS Statistics version 18 (SPSS, Inc., Chicago, IL, USA). All data in this study were normally distributed continuous data and are presented as mean ±  standard deviation (SD). Independent samples Kruskal-Wallis test was used to compare the results of each parameter among the 3 study groups. A *p*-value less than 0.05 was considered to be statistically significant for all tests.

## Results

### Phenotypic analysis of progenitor cells, T regulatory cells, and M2 macrophages

The mean percentage of CD34 + cells in the QQ-MNCs and PB-MNCs groups was 13.43 ± 4.89 and 5.70 ± 1.97 percent, respectively. The QQ-MNCs group had a significantly higher percentage of CD34 + cells than the PB-MNCs group (*p* = 0.047). The mean percentage of CD34 + CD133 + cells in the QQ-MNCs and PB-MNCs groups was 14.12 ± 7.98 and 1.92 ± 2.02 percent, respectively. The mean percentage of CD34 + CD133 + cells in the QQ-MNCs group was significantly higher than in the PB-MNCs group (*p* = 0.041). The mean percentage of CD206 + cells in the QQ-MNCs and PB-MNCs groups was 39.38 ± 30.26 and 11.70 ± 12.18 percent, respectively. Even though there was no statistically significant difference between groups, the percentage of CD206 + cells was higher in the QQ-MNCs group (*p* = 0.140). The mean percentage of CD4 + CD25 + CD127 + cells was non-significantly higher in the PB-MNCs group than in the QQ-MNCs group (7.38 ± 6.75 *vs.* 2.07 ± 2.60 percent, respectively; *p* = 0.347) (Table 1).

**Table 1:** Outcomes of *in vitro* study compared between the QQ-MNCs and PB-MNCs groups.

Evaluated parameters	QQ-MNCs group	PB-MNCs group	*p*-value
CD34 + cells (%)	13.43 ± 4.89	5.70 ± 1.97	***0.047***
CD34 + CD133 + cells (%)	14.12 ± 7.98	1.92 ± 2.02	***0.041***
CD206 + cells (%)	39.38 ± 30.26	11.70 ± 12.18	0.140
CD4 + CD25 + CD127 + cells (%)	2.07 ± 2.60	7.38 ± 6.75	0.347
CFU count (2 × 10^5^ cells/dish)	7.30 ± 4.60	1.60 ± 0.80	0.100

Data presented as mean ±  standard deviation

A *p*-value < 0.05 indicates statistical significance

Abbreviations: CD, cluster of differentiation; CFU, colony-forming unit; PB-MNCs, peripheral blood mononuclear cells; QQ-MNCs, Quality and Quantity mononuclear cells

### Endothelial progenitor cell colony formation assay (EPC-CFA)

There was no statistically significant difference in the mean colony-forming unit (CFU) count between the QQ-MNCs and PB-MNCs groups (7.3 ± 4.6 *vs.* 1.6 ± 0.8 CFUs, respectively; *p* = 1.00) (Table 1).

### Functional assessment

#### Sciatic Nerve Functional Index (SFI).

At 12-weeks post operation, the mean SFI score in the QQ-MNCs, PB-MNCs, and PBS groups was -39.30 ± 17.67, -39.35 ± 13.73, and -51.85 ± 10.30, respectively. The QQ-MNCs and PB-MNCs groups both had a higher SFI score than the PBS group, but the difference between groups was not statistically significant for either comparison (Table 2).

**Table 2:** Outcomes of functional assessment, electrophysiologic assessment, and wet-weight muscle examination.

Assessment parameters	Mean ± SD			*p*-value		
**PBS**	**PB-MNCs**	**QQ-MNCs**	**PBS** *vs.* **PB-MNCs**	**PBS** *vs.* **QQ-MNCs**	**PB-MNCs** *vs.* **QQ-MNCs**
**Functional assessment**						
- SFI	-51.85 ± 10.30	-39.35 ± 13.73	-39.30 ± 17.67	0.224	0.221	1.000
- BMS	6.33 ± 0.87	6.56 ± 0.88	7.44 ± 0.88	0.664	***0.018***	0.053
**Electrophysiologic assessment**						
- NCV (m/s)	12.00 ± 8.57	19.12 ± 11.70	16.93 ± 12.74	0.553	0.730	1.000
- Threshold (mV)	1297.50 ± 256.73	1269.58 ± 1141.19	988.54 ± 1016.82	0.385	0.898	1.000
**Wet weight muscle examination**						
- Muscle retention (%)	83.46 ± 5.72	86.34 ± 9.75	90.79 ± 6.18	1.000	0.142	0.651

Data presented as mean ±  standard deviation

A *p*-value < 0.05 indicates statistical significance

Abbreviations: BMS, Basso Mouse Scale; m/s, meter per second; mV, millivolts; NCV, nerve conduction velocity; PB-MNCs, peripheral blood mononuclear cells; PBS, phosphate buffered saline; QQ-MNCs, Quality and Quantity mononuclear cells; SFI, Sciatic Nerve Functional Index; SD, standard deviation; _vs._, versus

#### Basso Mouse Scale (BMS).

The mean BMS score in the QQ-MNCs, PB-MNCs, and PBS groups was 7.44 ± 0.88, 6.56 ± 0.88, and 6.33 ± 0.87, respectively. The QQ-MNCs group showed the highest BMS score, which was significantly higher than the BMS score in the PBS group (*p* = 0.018) (Table 2).

### Electrophysiologic examination

The mean nerve conduction velocity (NCV) outcome for the QQ-MNCs, PB-MNCs, and PBS groups was 16.93 ± 12.74, 19.12 ± 11.70, and 12.00 ± 8.57 meter/second (m/s), respectively. Although the results from both experimental groups were better than that from the PBS group, no statistically significant differences were observed for any of the 3 possible between group comparisons.

The mean threshold stimulation outcome for the QQ-MNCs, PB-MNCs, and PBS groups was 988.54 ± 1016.82, 1269.58 ± 1141.19, and 1297.50 ± 256.73 millivolts (mV), respectively. QQ-MNCs conferred the best result, but no significant differences were observed for any of the 3 between group comparisons (Table 2).

### Histomorphologic assessment

#### Nerve specimens.

Histomorphologic testing of nerve specimens included measurement of axon fiber diameter (cross-sectional specimen), measurement of myelin thickness, and recording the percentage of nerve density per area of investigation. All proximal and distal nerve specimens were sectioned and stained with H&E stain and mGT stain.

#### Proximal nerve specimens.

**Fig 2:**
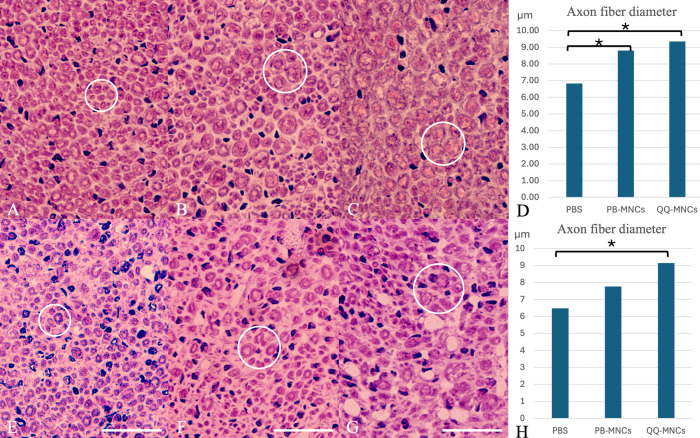
Cross-sectional study of proximal nerve specimens and distal nerve specimens stained with hematoxylin and eosin (H&E) stain. Row 1, proximal nerve specimens submitted to (A) Phosphate buffered saline (PBS) group; (B) Peripheral blood mononuclear cells (PB-MNCs) group; and, (C) Quality and Quantity mononuclear cells (QQ-MNCs) group. This figure shows that the QQ-MNCs group had a significantly larger axon fiber diameter than the other groups. The white circles indicate the cross-sectional surface of the axon fiber in each group. (D) The mean axon fiber diameter (μm, micrometer) in each group. Row 2, distal nerve specimens submitted to (E) Phosphate buffered saline (PBS) group; (F) Peripheral blood mononuclear cells (PB-MNCs) group; and, (G) Quality and Quantity mononuclear cells (QQ-MNCs) group. This figure shows that the QQ-MNCs group had a significantly larger axon fiber diameter than PBS group. (H) The mean axon fiber diameter (μm) in each group. The white circles indicate the cross-sectional surface of the axon fiber in each group. (Bar = 20 μm) An asterisk (*) indicates statistical significance between groups (*p* < 0.05).

The mean diameter of nerve fiber (μm, micrometer) in the QQ-MNCs, PB-MNCs, and PBS groups was 9.35 ± 0.86, 8.81 ± 0.77, and 6.83 ± 0.74 μm, respectively. The QQ-MNCs group had the largest axon fiber diameter, which was significantly greater than the mean axon fiber diameter in the PBS group (*p* < 0.001). There was no statistically significant difference in the axon fiber diameter between the QQ-MNCs and PB-MNCs groups (Fig 2).

**Fig 3:**
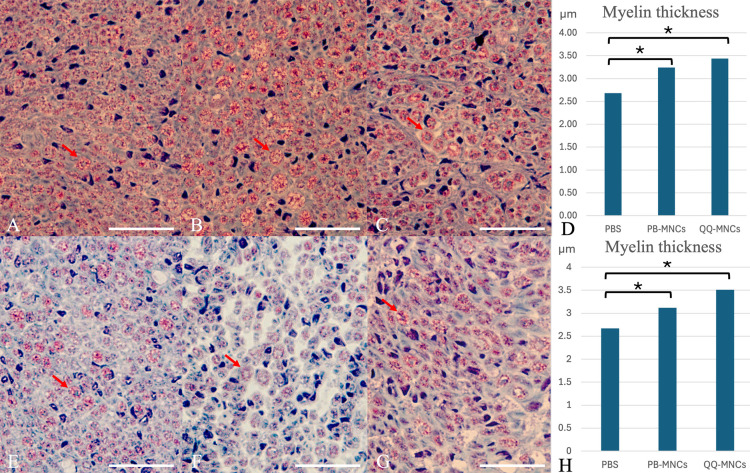
Cross-sectional study of proximal nerve specimens and distal nerve specimens stained with modified Gomori trichome (mGT) stain to demonstrate the myelin thickness in nerve fibers. Row 1, proximal nerve specimens submitted to (A) Phosphate buffered saline (PBS) group; (B) Peripheral blood mononuclear cells (PB-MNCs) group; and, (C) Quality and Quantity mononuclear cells (QQ-MNCs) group. This figure shows that the QQ-MNCs group had significantly thicker of myelin than the other groups. (D) The mean of myelin thickness (μm, micrometer) in each group. Row 2, distal nerve specimens submitted to (E) Phosphate buffered saline (PBS) group; (F) Peripheral blood mononuclear cells (PB-MNCs) group; and, (G) Quality and Quantity mononuclear cells (QQ-MNCs) group. This figure shows that the QQ-MNCs group had significantly thicker of myelin than the other groups. (H) The mean myelin thickness (μm) in each group. The red arrows indicate myelin in nerve fiber which stain in red color. (Bar = 20 μm) An asterisk (*) indicates statistical significance between groups (*p* < 0.05).

The mean thickness of myelin in nerve fiber (μm) in the QQ-MNCs, PB-MNCs, and PBS groups was 3.44 ± 0.18, 3.24 ± 0.19, and 2.68 ± 0.25 μm, respectively. The QQ-MNCs group had the greatest myelin thickness, which was significantly greater than the myelin thickness in the PBS group (*p* < 0.001). There was no statistically significant difference in myelin thickness between the QQ-MNCs group and the PB-MNCs group (Fig 3).

The mean nerve density percentage in the QQ-MNCs, PB-MNCs, and PBS groups was 77.09 ± 6.53, 68.45 ± 3.25, and 57.29 ± 9.15 percent, respectively. The QQ-MNCs group had the highest nerve density percentage. There was a significant difference in nerve density percentage between the PB-MNCs and PBS groups (*p* < 0.001), and between the QQ-MNCs and PB-MNCs groups (*p* = 0.049) (Table 3).

**Table 3:** Histomorphologic studies of proximal nerve, distal nerve, and gastrocnemius muscle specimens.

Histomorphologic parameters	Mean ± SD			*p*-value		
**PBS**	**PB-MNCs**	**QQ-MNCs**	**PBS** *vs.* **PB-MNCs**	**PBS** *vs.* **QQ-MNCs**	**PB-MNCs** *vs.* **QQ-MNCs**
**Proximal nerve**						
- Nerve fiber diameter (μm)	6.83 ± 0.74	8.81 ± 0.77	9.35 ± 0.86	***0.014***	***<0.001***	0.725
- Myelin thickness (μm)	2.68 ± 0.25	3.24 ± 0.19	3.44 ± 0.18	***0.015***	***<0.001***	0.442
- Nerve density (%)	57.29 ± 9.15	68.45 ± 3.25	77.09 ± 6.53	0.174	***<0.001***	***0.049***
**Distal nerve**						
- Nerve fiber diameter (μm)	6.48 ± 0.64	7.76 ± 1.05	9.15 ± 0.64	0.163	***<0.001***	0.058
- Myelin thickness (μm)	2.67 ± 0.19	3.12 ± 0.18	3.51 ± 0.31	***0.035***	***<0.001***	0.207
- Nerve density (%)	58.91 ± 18.50	75.47 ± 11.77	79.10 ± 8.75	0.697	0.070	1.000
**Gastrocnemius muscle**						
- Muscle fiber diameter (μm)	63.28 ± 4.20	75.06 ± 7.77	77.83 ± 7.93	***0.008***	***0.002***	1.000
- Vessel-to-muscle ratio	0.81 ± 0.08	0.90 ± 0.18	1.10 ± 0.23	0.799	***0.004***	0.088

Data presented as mean ±  standard deviation

A *p*-value < 0.05 indicates statistical significance

Abbreviations: PB-MNCs, peripheral blood mononuclear cells; PBS, phosphate buffered saline; QQ-MNCs, Quality and Quantity mononuclear cells; SD, standard deviation; *vs.*, versus; μm, micrometers

#### Distal nerve specimens.

The mean axon fiber diameter (μm) for the QQ-MNCs, PB-MNCs, and PBS groups was 9.15 ± 0.64, 7.76 ± 1.05, and 6.48 ± 0.64 μm, respectively. The QQ-MNCs group had the largest axon fiber diameter, which was also significantly larger than the axon fiber diameter in the PBS group (*p* < 0.001). There was no significant difference in the axon fiber diameter between the PB-MNCs and PBS groups (Fig 2).

In the QQ-MNCs, PB-MNCs, and PBS groups, the mean thickness of myelin in nerve fiber (μm) was 3.51 ± 0.31, 3.12 ± 0.18, and 2.67 ± 0.19 μm, respectively. The QQ-MNCs group had the greatest thickness, which was also significantly different from the PBS group (*p* < 0.001) (Fig 3).

The mean nerve density percentage in the QQ-MNCs, PB-MNCs, and PBS groups was 79.10 ± 8.75, 75.47 ± 11.77, and 58.91 ± 18.50 percent, respectively. The QQ-MNCs group had the greatest nerve density percentage among the 3 groups. There was no statistically significant difference between groups for any of the 3 possible group comparisons (all *p* > 0.05) (Table 3).

#### Muscle specimens.

Histomorphologic testing of gastrocnemius muscle specimens comprised measurements of the cross-sectional diameter of the muscle, and vessel-to-muscle ratio. Muscle specimens were stained with H&E and mGT and then evaluated using the ImageJ software program.

The mean diameter of muscle fiber (μm) in the QQ-MNCs, PB-MNCs, and PBS groups was 77.83 ± 7.93, 75.06 ± 7.77, and 63.28 ± 4.20 μm, respectively. The QQ-MNCs group had the largest muscle fiber diameter, which was also significantly larger than the mean muscle fiber diameter in the PBS group (*p* = 0.002). There was no significant difference between the QQ-MNCs and PB-MNCs groups.

In the QQ-MNCs, PB-MNCs, and PBS groups, the mean vessel-to-muscle ratio was 1.10 ± 0.23, 0.90 ± 0.18, and 0.81 ± 0.08, respectively. The QQ-MNCs group had the highest ratio, which was also significantly different from that observed in the PBS group (*p* = 0.004). Similar to the mean muscle fiber measurement, there was no significant difference between the QQ-MNCs and PB-MNCs groups (Table 3).

### Immunohistochemistry testing

#### Proximal nerve specimens.

The mean result from immunohistochemistry staining (IOD, integrated optical density) of S100 in the QQ-MNCs, PB-MNCs, and PBS groups was 2.97 ± 1.18, 2.17 ± 0.90, and 0.75 ± 0.54 IOD, respectively. The results in the QQ-MNCs and PBMNCs groups were both significantly different from the PBS group value (*p* = 0.001 and *p* = 0.03, respectively), but no significant difference between the QQ-MNCs and PB-MNCs groups was observed.

For SOX10, the mean result in the QQ-MNCs, PB-MNCs, and PBS groups was 5.68 ± 4.26, 2.77 ± 0.94, and 1.06 ± 0.29 IOD, respectively. The results in the QQ-MNCs and PBMNCs groups were both significantly different from the PBS group value (*p* < 0.001 and *p* = 0.007, respectively); however, no significant difference was found between the QQ-MNCs and PB-MNCs groups.

Concerning GFAP, the mean result in the QQ-MNCs, PB-MNCs, and PBS groups was 3.84 ± 1.97, 2.54 ± 1.07, and 0.82 ± 0.45 IOD, respectively. The results in the QQ-MNCs and PB-MNCs groups were both significantly different from the PBS group result (*p* < 0.001 and *p* = 0.009, respectively), but there was no significant difference between the QQ-MNCs and PB-MNCs groups.

Regarding neurofilament, the mean result in the QQ-MNCs, PB-MNCs, and PBS groups was 21.04 ± 4.07, 25.32 ± 6.40, and 8.62 ± 4.37 IOD, respectively. The results in the QQ-MNCs and PBMNCs groups were both significantly different from the PBS group value (*p* < 0.001 and *p* = 0.008, respectively), but no significant difference between the QQ-MNCs and PB-MNCs groups was found.

For IL-1β, the mean result in the QQ-MNCs, PB-MNCs, and PBS groups was 0.43 ± 0.34, 0.61 ± 0.53, and 3.43 ± 2.56 IOD, respectively. The QQ-MNCs group result was significantly different from both the PB-MNCs and PBS groups (*p* = 0.01 and *p* = 0.001, respectively); however, there was no significant difference between the PB-MNCs and PBS groups (*p* = 1.00).

For VEGF, the mean result in the QQ-MNCs, PB-MNCs, and PBS groups was 23.10 ± 6.94, 17.61 ± 5.07, and 11.31 ± 2.36 IOD, respectively. The results in the QQ-MNCs and PB-MNCs groups were both significantly different from the PBS group result (*p* = 0.001 and *p* = 0.045, respectively), but there was no significant difference between the QQ-MNCs and PB-MNCs groups.

Concerning anti-HNA, the mean result in the QQ-MNCs, PB-MNCs, and PBS groups was 0.41 ± 0.21, 0.54 ± 0.36, and 0.00 ± 0.00 IOD, respectively. The results in the QQ-MNCs and PB-MNCs groups were both significantly different from that found in the PBS group (*p* < 0.001 and *p* = 0.015, respectively), but there was no significant difference between the QQ-MNCs and PB-MNCs groups.

For TNF-α, the mean result in the QQ-MNCs, PB-MNCs, and PBS groups was 0.41 ± 0.21, 0.53 ± 0.29, and 2.04 ± 1.41 IOD, respectively. The results in the QQ-MNCs and PB-MNCs groups were both significantly different from the PBS group value (*p* = 0.001 and *p* = 0.011 respectively), but there was no significant difference between the QQ-MNCs and PB-MNCs groups.

**Fig 4:**
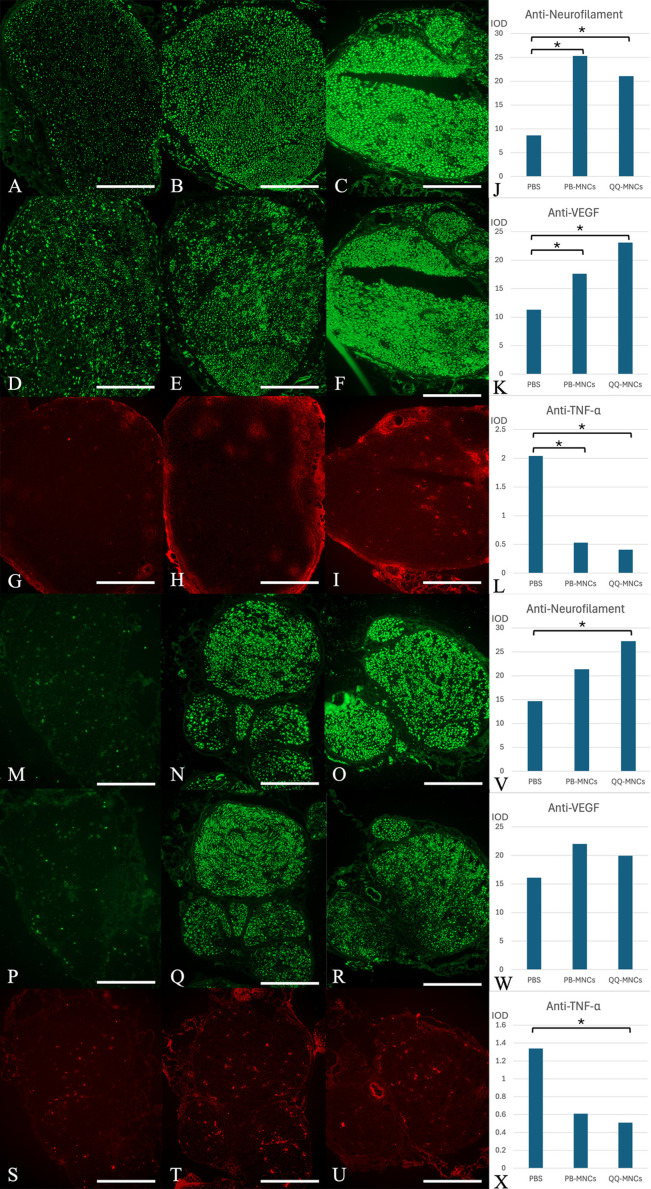
Cross-sectional study of nerve specimens stained with immunohistochemistry staining. Row 1-3, proximal nerve specimens submitted to (A), (D) and (G) Phosphate buffered saline (PBS) group; (B), (E) and (H) Peripheral blood mononuclear cells (PB-MNCs) group; and, (C), (F) and (I) Quality and Quantity mononuclear cells (QQ-MNCs) group, stained with anti-neurofilament (row 1), anti-vascular endothelial growth factor (anti-VEGF) antibody (row 2) and anti-tumor necrosis factor alpha (anti-TNF-α) antibody (row 3), respectively. (J), (K), (L) The mean of integrated optical density (IOD) of anti-neurofilament, anti-VEGF, and anti-TNF-α, respectively. Row 4-6, distal nerve specimens submitted to (M), (P) and (S) Phosphate buffered saline (PBS) group; (N), (Q) and (T) Peripheral blood mononuclear cells (PB-MNCs) group; and, (O), (R) and (U) Quality and Quantity mononuclear cells (QQ-MNCs) group, stained with anti-neurofilament (row 4), anti-vascular endothelial growth factor (anti-VEGF) antibody (row 5) and anti-tumor necrosis factor alpha (anti-TNF-α) antibody (row 6), respectively. (V), (W), (X) The mean integrated optical density (IOD) of anti-neurofilament, anti-VEGF, and anti-TNF-α, respectively. (Bar = 50 μm) An asterisk (*) indicates statistical significance between groups (*p* < 0.05).

**Fig 5:**
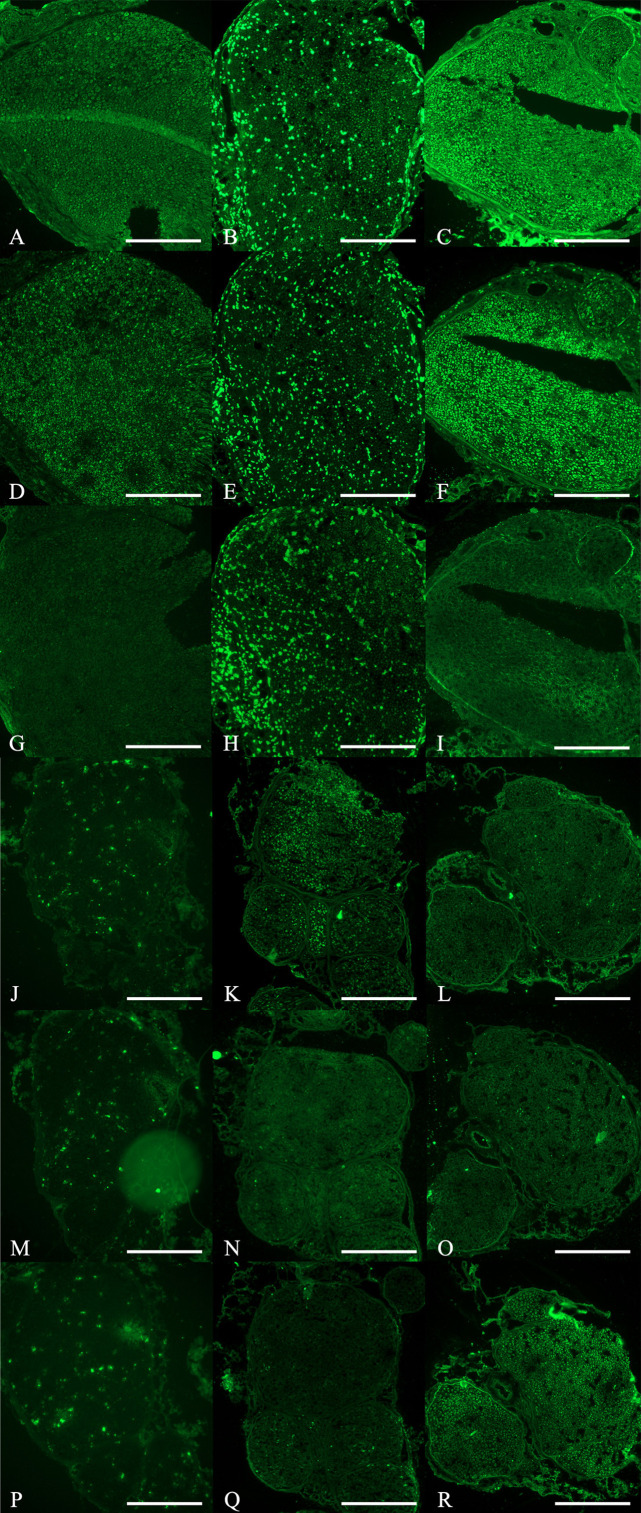
Cross-sectional study of nerve specimens stained with immunohistochemistry staining. Row 1-3, proximal nerve specimens submitted to (A), (D) and (G) Phosphate buffered saline (PBS) group; (B), (E) and (H) Peripheral blood mononuclear cells (PB-MNCs) group; and, (C), (F) and (I) Quality and Quantity mononuclear cells (QQ-MNCs) group, stained with anti-S100 alpha antibody (anti-S100) (row 1), anti-SOX10 antibody (anti-SOX10) (row 2) and anti-GFAP antibody (anti-GFAP) (row 3), respectively. Row 4-6, distal nerve specimens submitted to (J), (M) and (P) Phosphate buffered saline (PBS) group; (K), (N) and (Q) Peripheral blood mononuclear cells (PB-MNCs) group; and, (L), (O) and (R) Quality and Quantity mononuclear cells (QQ-MNCs) group, stained with anti-S100 alpha antibody (anti-S100) (row 4), anti-SOX10 antibody (anti-SOX10) (row 5) and anti-GFAP antibody (anti-GFAP) (row 6), respectively. (Bar = 50 μm).

**Fig 6:**
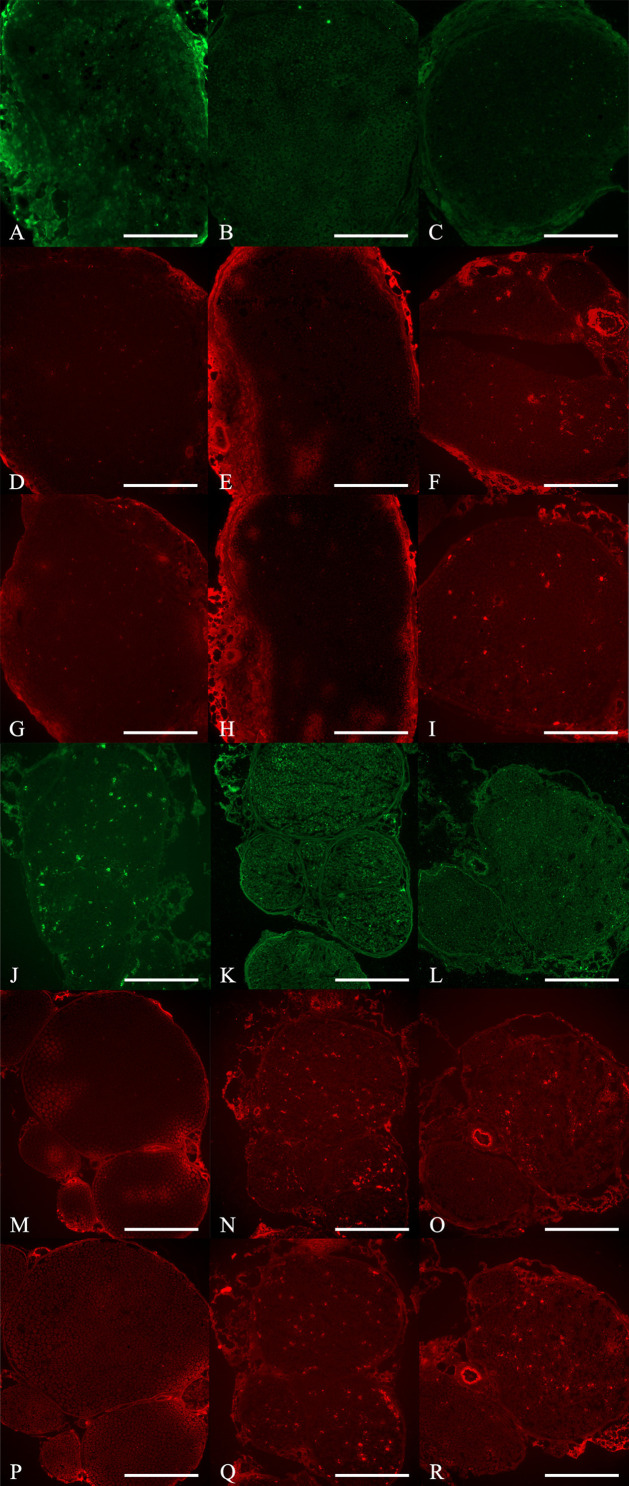
Cross-sectional study of nerve specimens stained with immunohistochemistry staining. Row 1-3, proximal nerve specimens submitted to (A), (D) and (G) Phosphate buffered saline (PBS) group; (B), (E) and (H) Peripheral blood mononuclear cells (PB-MNCs) group; and, (C), (F) and (I) Quality and Quantity mononuclear cells (QQ-MNCs) group, stained with anti-IL-1 beta antibody (anti-IL-1β) (row 1), anti-human nuclear antigen antibody (Anti-HNA) (row 2) and anti-von Willebrand factor antibody (anti-vWF) (row 3), respectively. Row 4-6, distal nerve specimens submitted to (J), (M) and (P) Phosphate buffered saline (PBS) group; (K), (N) and (Q) Peripheral blood mononuclear cells (PB-MNCs) group; and, (L), (O) and (R) Quality and Quantity mononuclear cells (QQ-MNCs) group, stained with anti-IL-1 beta antibody (anti-IL-1β) (row 4), anti-human nuclear antigen antibody (Anti-HNA) (row 5) and anti-von Willebrand factor antibody (anti-vWF) (row 6), respectively. (Bar = 50 μm).

For vWF, the mean result in the QQ-MNCs, PB-MNCs, and PBS groups was 0.55 ± 0.28, 0.48 ± 0.28, and 0.00 ± 0.00 IOD, respectively. The results in the QQ-MNCs and PB-MNCs groups were both significantly different from that observed in the PBS group (*p* < 0.001 and *p* = 0.003, respectively); however, there was no significant difference between the QQ-MNCs and PB-MNCs groups (Figs 4-6, Table 4).

**Table 4:** Immunohistochemistry outcomes of proximal nerve specimens.

Immunohistochemistry Parameters (IOD)	Mean ± SD			*p*-value		
**PBS**	**PB-MNCs**	**QQ-MNCs**	**PBS** *vs.* **PB-MNCs**	**PBS** *vs.* **QQ-MNCs**	**PB-MNCs** *vs.* **QQ-MNCs**
S100	0.75 ± 0.54	2.17 ± 0.90	2.97 ± 1.18	***0.03***	***0.001***	0.688
SOX10	1.06 ± 0.29	2.77 ± 0.94	5.68 ± 4.26	***0.007***	***<0.001***	1.00
GFAP	0.82 ± 0.45	2.54 ± 1.07	3.84 ± 1.97	***0.009***	***<0.001***	0.842
Neurofilament	8.62 ± 4.37	25.32 ± 6.40	21.04 ± 4.07	***0.008***	***<0.001***	0.928
IL-1β	3.43 ± 2.56	0.61 ± 0.53	0.43 ± 0.34	1.00	***0.001***	***0.01***
VEGF	11.31 ± 2.36	17.61 ± 5.07	23.10 ± 6.94	***0.045***	***0.001***	0.587
Anti-HNA	0.0 ± 0.0	0.54 ± 0.36	0.41 ± 0.21	***0.015***	***<0.001***	0.376
TNF-α	2.04 ± 1.41	0.53 ± 0.29	0.41 ± 0.21	***0.011***	***0.001***	1.00
vWF	0.0 ± 0.0	0.48 ± 0.28	0.55 ± 0.28	***0.003***	***<0.001***	1.00

Data presented as mean ±  standard deviation

A *p*-value < 0.05 indicates statistical significance

Abbreviations: anti-HNA; anti-human nuclear antigen; GFAP, glial fibrillary acid protein; IL-1β, interleukin-1 beta; IOD, integrated optical density; PB-MNCs, peripheral blood mononuclear cells; PBS, phosphate buffered saline; QQ-MNCs, Quality and Quantity mononuclear cells; S100, S100 proteins; SD, standard deviation; SOX10, SOX10 proteins; TNF-α; tumor necrosis factor-alpha; VEGF, vascular endothelial growth factor; *vs*., versus; vWF, von-Willebrand factor

#### Distal nerve specimens.

The mean result from immunohistochemistry staining of S100 in the QQ-MNCs, PB-MNCs, and PBS groups was 1.73 ± 2.06, 0.78 ± 0.94, and 0.29 ± 0.17 IOD, respectively. The QQ-MNCs group had a significantly higher value compared to that found in the PBS group (*p* = 0.021). In contrast, no significant differences were observed between the two experimental groups, or between the PB-MNCs and PBS groups (Fig 5).

For SOX10, the mean result in the QQ-MNCs, PB-MNCs, and PBS groups was 2.06 ± 2.38, 2.87 ± 1.95, and 1.06 ± 0.99 IOD, respectively. There were no significant differences between groups for any of the three possible group comparisons (Fig 5).

Regarding GFAP, the mean result in the QQ-MNCs, PB-MNCs, and PBS groups was 2.98 ± 2.11, 1.27 ± 0.90, and 0.30 ± 0.16 IOD, respectively. The results in the QQ-MNCs and PB-MNCs groups were both significantly different from the PBS group value (*p* < 0.001 and *p* = 0.02, respectively). In contrast, there was no significant difference observed between the QQ-MNCs and PB-MNCs groups (Fig 5).

For neurofilament, the mean result in the QQ-MNCs, PB-MNCs, and PBS groups was 27.25 ± 5.19, 21.33 ± 7.28, and 14.69 ± 3.61 IOD, respectively. The QQ-MNCs group result was significantly different from the PBS group result (*p* = 0.002). However, there were no significant differences observed between the two experimental groups, or between the PB-MNCs and PBS groups (Fig 4).

Concerning IL-1β, the mean result in the QQ-MNCs, PB-MNCs, and PBS groups was 0.47 ± 0.51, 0.51 ± 0.70, and 1.74 ± 3.15 IOD, respectively. There were no significant differences between groups for any of the three possible group comparisons (Fig 6).

For VEGF, the mean result in the QQ-MNCs, PB-MNCs, and PBS groups was 19.96 ± 7.07, 22.02 ± 8.06, and 16.11 ± 4.10 IOD, respectively. There were no significant differences between groups for any of the three possible group comparisons (Fig 4).

For anti-HNA, the mean result in the QQ-MNCs, PB-MNCs, and PBS groups was 1.03 ± 0.31, 0.61 ± 0.43, and 0.00 ± 0.00 IOD, respectively. The results in the QQ-MNCs and PBMNCs groups were both significantly different from the PBS group value (*p* < 0.001 and *p* = 0.014, respectively), but there was no significant difference between the two experimental groups (Fig 6).

Regarding TNF-α, the mean result in the QQ-MNCs, PB-MNCs, and PBS groups was 0.51 ± 0.35, 0.61 ± 0.29, and 1.34 ± 1.18 IOD, respectively. The QQ-MNCs group showed a significant difference from the PBS group (*p* = 0.018); however, there were no significant differences observed between the two experimental groups, or between the PB-MNCs and PBS groups (Fig 4).

For vWF, the mean result in the QQ-MNCs, PB-MNCs, and PBS groups was 0.89 ± 0.45, 0.63 ± 0.34, and 0.00 ± 0.00 IOD, respectively. The results in the QQ-MNCs and PB-MNCs groups were both significantly different from the PBS group result (*p* < 0.001 and *p* = 0.004, respectively), but no significant difference was found between the QQ-MNCs and PB-MNCs groups (Fig 6, Table 5).

**Table 5:** Immunohistochemistry outcomes of distal nerve specimens.

Immunohistochemistry parameters (IOD)	Mean ± SD			*p*-value		
**PBS**	**PB-MNCs**	**QQ-MNCs**	**PBS** *vs.* **PB-MNCs**	**PBS** *vs.* **QQ-MNCs**	**PB-MNCs** *vs.* **QQ-MNCs**
S100	0.29 ± 0.17	0.78 ± 0.94	1.73 ± 2.06	0.592	***0.021***	0.442
SOX10	1.06 ± 0.99	2.87 ± 1.95	2.06 ± 2.38	0.1	0.1	0.1
GFAP	0.30 ± 0.16	1.27 ± 0.90	2.98 ± 2.11	***0.02***	***<0.001***	0.652
Neurofilament	14.69 ± 3.61	21.33 ± 7.28	27.25 ± 5.19	0.263	***0.002***	0.222
IL-1β	1.74 ± 3.15	0.51 ± 0.70	0.47 ± 0.51	0.072	0.072	0.072
VEGF	16.11 ± 4.10	22.02 ± 8.06	19.96 ± 7.07	0.211	0.211	0.211
Anti-HNA	0.0 ± 0.0	0.61 ± 0.43	1.03 ± 0.31	***0.014***	***<0.001***	0.4
TNF-α	1.34 ± 1.18	0.61 ± 0.29	0.51 ± 0.35	0.15	***0.018***	1.00
vWF	0.0 ± 0.0	0.63 ± 0.34	0.89 ± 0.45	***0.004***	***<0.001***	1.00

Data presented as mean ±  standard deviation

A *p*-value < 0.05 indicates statistical significance

Abbreviations: anti-HNA; anti-human nuclear antigen; GFAP, glial fibrillary acid protein; IL-1β, interleukin-1 beta; IOD, integrated optical density; PB-MNCs, peripheral blood mononuclear cells; PBS, phosphate buffered saline; QQ-MNCs, Quality and Quantity mononuclear cells; S100, S100 proteins; SD, standard deviation; SOX10, SOX10 proteins; TNF-α; tumor necrosis factor-alpha; VEGF, vascular endothelial growth factor; *vs*., versus; vWF, von-Willebrand factor

#### Gastrocnemius muscle.

The mean result from immunohistochemistry staining of IL-1β in the QQ-MNCs, PB-MNCs, and PBS groups was 0.75 ± 0.24, 0.87 ± 0.58, and 1.96 ± 0.58 IOD, respectively. The results in the QQ-MNCs and PB-MNCs groups were both significantly different from the result in the PBS group (*p* = 0.006 and *p* = 0.005, respectively), but there was no significant difference between the QQ-MNCs and PB-MNCs groups.

Regarding VEGF, the mean result in the QQ-MNCs, PB-MNCs, and PBS groups was 2.71 ± 0.77, 2.45 ± 1.30, and 1.30 ± 0.33 IOD, respectively. The results in the QQ-MNCs and PB-MNCs groups were both significantly different from the PBS group result (*p* = 0.002 and *p* = 0.048, respectively), but no significant difference between the experimental groups was observed.

For TNF-α, the mean result in the QQ-MNCs, PB-MNCs, and PBS groups was 0.55 ± 0.19, 0.89 ± 0.57, and 1.25 ± 0.82 IOD, respectively. The QQ-MNCs group showed a significant difference compared to the PBS group (*p* = 0.043). However, there were no significant differences observed between the two experimental groups, or between the PB-MNCs and PBS groups.

**Fig 7:**
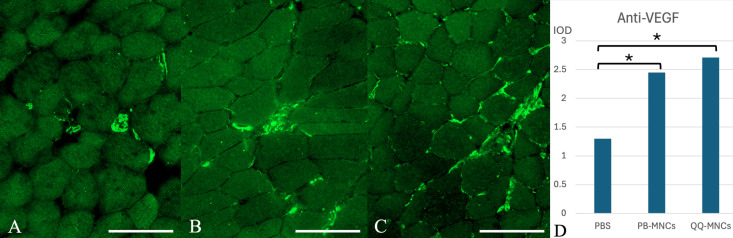
Cross-sectional study of gastrocnemius muscle specimens stained with anti-vascular endothelial growth factor (anti-VEGF) antibody. (A) Phosphate buffered saline (PBS) group; (B) Peripheral blood mononuclear cells (PB-MNCs) group; and, (C) Quality and Quantity mononuclear cells (QQ-MNCs) group. This figure shows that the QQ-MNCs group had significantly more positive staining than the other groups, which indicates a higher rate of angiogenesis. (Bar =  50 μm) (D) The mean integrated optical density (IOD) of anti-VEGF. An asterisk (*) indicates statistical significance between groups (*p* < 0.05).

**Fig 8:**
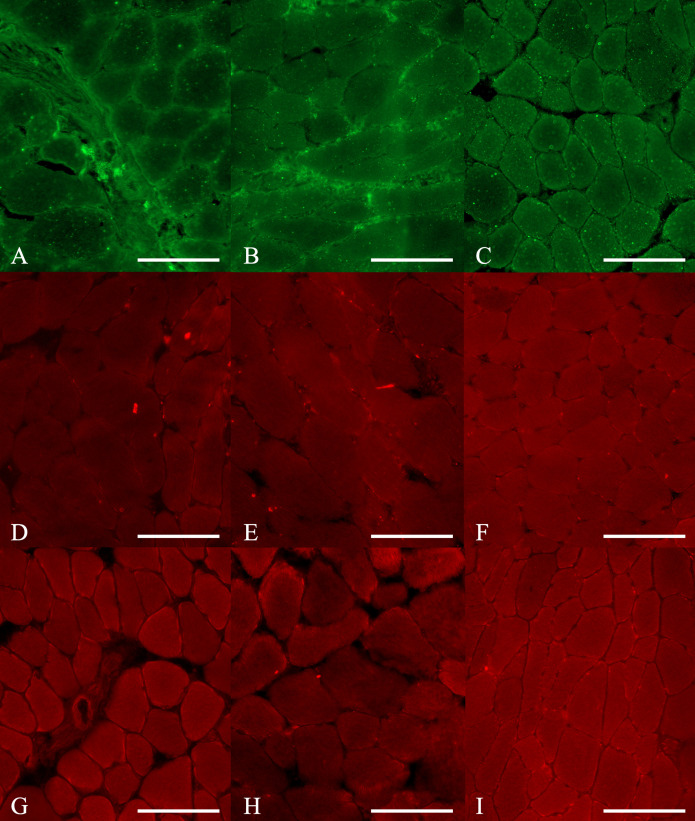
Cross-sectional study of gastrocnemius muscle specimens stained with immunohistochemistry staining. Gastrocnemius muscle specimens submitted to (A), (D) and (G) Phosphate buffered saline (PBS) group; (B), (E) and (H) Peripheral blood mononuclear cells (PB-MNCs) group; and, (C), (F) and (I) Quality and Quantity mononuclear cells (QQ-MNCs) group, stained with anti-IL-1 beta antibody (anti-IL-1β) (row 1), anti-tumor necrosis factor alpha (anti-TNF- α) antibody (row 2) and anti-von Willebrand factor antibody (anti-vWF) (row 3), respectively. (Bar = 50 μm).

Concerning vWF, the mean result in the QQ-MNCs, PB-MNCs, and PBS groups was 0.96 ± 0.49, 0.70 ± 0.36, and 0.00 ± 0.00 IOD, respectively. The results in the QQ-MNCs and PB-MNCs groups were both significantly different from the PBS group result (*p* < 0.001 and *p* = 0.005, respectively). No significant difference between the QQ-MNCs and PB-MNCs groups was found (Figs 7 and 8, S1 and S2 Figs, Table 6).

**Table 6:** Immunohistochemistry outcomes of gastrocnemius muscle specimens.

Immunohistochemistry parameters (IOD)	Mean ± SD			*p*-value		
**PBS**	**PB-MNCs**	**QQ-MNCs**	**PBS** *vs.* **PB-MNCs**	**PBS** *vs.* **QQ-MNCs**	**PB-MNCs** *vs.* **QQ-MNCs**
IL-1β	1.96 ± 0.58	0.87 ± 0.58	0.75 ± 0.24	***0.005***	***0.006***	1.00
VEGF	1.30 ± 0.33	2.45 ± 1.30	2.71 ± 0.77	***0.048***	***0.002***	0.884
TNF-α	1.25 ± 0.82	0.89 ± 0.57	0.55 ± 0.19	0.886	***0.043***	0.443
vWF	0.0 ± 0.0	0.70 ± 0.36	0.96 ± 0.49	***0.005***	***<0.001***	1.00

Data presented as mean ±  standard deviation

A *p*-value < 0.05 indicates statistical significance

Abbreviations: anti-HNA; anti-human nuclear antigen; IL-1β, interleukin-1 beta; IOD, integrated optical density; PB-MNCs, peripheral blood mononuclear cells; PBS, phosphate buffered saline; QQ-MNCs, Quality and Quantity mononuclear cells; SD, standard deviation; TNF-α; tumor necrosis factor-alpha; VEGF, vascular endothelial growth factor; *vs.*, versus; vWF, von-Willebrand factor

### Gastrocnemius wet-weight muscle

The mean gastrocnemius muscle retention percentage in the QQ-MNCs, PB-MNCs, and PBS groups was 90.79 ± 6.18, 86.34 ± 9.75, and 83.46 ± 5.72 percent, respectively. The QQ-MNCs group showed the highest percentage retention of wet-weight gastrocnemius muscle mass at 12-weeks post operation, but no statistically significant difference was observed between groups for any of the 3 possible group comparisons (Table 2).

## Discussion

The adverse consequences of peripheral nerve injuries are almost always life-altering and are often devastating. These injuries manifest as abnormal communication between the brain and its target organs [31]. Delayed recovery can lead to long-term disability, which often adversely affects various aspects of an individual’s life, including behavior, mobility, perception, consciousness, and sensations of skin and joints [32, 33]. Well-planned nerve repair and appropriate strategies to promote nerve regeneration and to shorten the recovery time are factors that heavily influence patient outcomes and quality of life. Mononuclear cells (MNCs), which are derived from peripheral venous blood, have demonstrated promising evidence of their ability to enhance nerve regeneration. Previous studies demonstrated the capability of MNCs to enhance the nerve regeneration process [4,5,9,11]. These cells act as a source of Schwann-type cells, and they maximize the intrinsic regenerative capacity via the production of growth factors, cytokines, and immunomodulation response. QQ-MNCs or MNCs cultured in Quality and Quantity (QQ) culture media increase the number of progenitor cells, have anti-inflammatory properties, and improve angiogenesis [12, 13]. To further assess QQ-MNCs and their ability to enhance peripheral nerve regeneration, the current study set forth to investigate the efficacy of QQ-MNCs for promoting nerve regeneration in a mouse sciatic nerve transection model.

Our results showed the levels of S100, SOX10, GFAP, and neurofilament to be significantly increased at the site of nerve injury in the QQ-MNCs group compared to the PB-MNCs and PBS groups. These findings suggest that the nerve regeneration process can be enhanced by QQ-MNCs. These observed outcomes correspond with those from previous studies that showed the regenerative properties of MNCs in nerve injury repair [4,5,9,11].

Histomorphologic studies and wet-weight gastrocnemius muscle examination showed that the QQ-MNCs group yielded the best results relative to size of axon fiber diameter, myelin thickness, percentage of nerve density, size of muscle diameter, vessel-to-muscle ratio, and the percentage of muscle retention. These outcomes suggest the efficacy of QQ-MNCs for maintaining nerve viability, enhancing the regenerative process, and reducing gastrocnemius muscle atrophy, which is the end organ supplied by the sciatic nerve.

In distal nerve specimens, the outcomes of QQ-MNCs were higher than the outcomes of PB-MNCs, but without statistical significance. However, the results in the QQ-MNCs group were better than in the other 2 groups for almost all histomorphologic and immunohistochemistry parameters (Tables 3 and 5, respectively). These results strongly suggest the superior ability of QQ-MNCs in nerve regeneration when compared to that of PB-MNCs.

In the functional assessment, the QQ-MNCs group exhibited superior results on the Basso Mouse Scale (BMS), which was significantly different from the mean BMS score in the PBS group. This finding that QQ-MNCs improve functional outcomes following nerve injury further supports the efficacy of QQ-MNCs in nerve regeneration.

Angiogenesis is a significant challenge in nerve repair and reconstruction. Achieving angiogenesis is essential for ensuring cell survival and preventing necrosis. It has been hypothesized that vascular endothelial cells play a vital role in guiding the regeneration of peripheral nerve axons via the production of vascular endothelial growth factor (VEGF), which initiates angiogenesis prior the restoration of damaged nerves [34]. VEGF and von Willebrand factor (vWF) serve as potential multifunctional cytokines that effectively stimulate the proliferation of Schwann cells and blood vessels, and that concurrently augment axonal outgrowth from the dorsal root ganglia [35, 36]. Our findings demonstrate that the QQ-MNCs group exhibited a notable increase in the levels of both VEGF and vWF around the nerve injury site. This suggests that QQ-MNCs may possess superior angiogenic properties compared to PB-MNCs and PBS.

Following nerve injury, the early stages of inflammation play a beneficial role by facilitating the removal of tissue debris and elevating levels of neurotrophic factors. However and in contrast, a persistent and prolonged inflammatory response influences the release of substantial amounts of inflammatory cytokines by inflammatory cells, which leads to further damage to the microenvironment, which is detrimental to nerve recovery and can compromise the health of unaffected nerves. Moreover, excessive inflammation impairs and obstructs the restoration of normal bodily functions following nerve injury. It has also been reported that persistent and unregulated inflammation is a primary instigator of a wide array of nerve pathologies, including neuropathic pain and autoimmune diseases [37,38]. TNF-α and IL-1β are proinflammatory cytokines that play a pivotal in regulating inflammatory responses after peripheral or central nervous system injury. At the lesion site, TNF-α expression increases, which influences substantial macrophage recruitment [39]. Previously reported experiments that were conducted *in vitro* demonstrated that TNF-α has a direct impact on Schwann cells, indicating that TNF-α leads to a reduction in Schwann cell proliferation in a manner that is dependent on the dosage of TNF-α [40,41]. Activation of IL-1β ultimately results in a sustained inflammatory response [42]. Research has established a close association between IL-1β and numerous inflammation-related conditions, including neurodegenerative diseases, chronic obstructive pulmonary disease, and rheumatoid arthritis [43,44]. Our *in vitro* experiment showed that QQ-MNCs can increase not only the percentage of progenitor cells, but also M2 macrophages and inactivated T regulatory cells (Table 1). These outcomes correspond with the marked decrease in TNF-α and IL-1β levels among QQ-MNCs group mice. This underscores the remarkable anti-inflammatory efficacy of QQ-MNCs, which was shown to be greater than that of both the PB-MNCs group and the PBS group.

In this study, we aimed to confirm both the engraftment of human stem cells (MNCs) in nude mice at 12-weeks after nerve transection and repair, and cell implantation by staining of all nerve specimens with anti-HNA. The results at 12 weeks revealed that nerve specimens in both experimental groups (QQ-MNCs and PB-MNCs) exhibited positive staining with anti-HNA. These findings confirm the viability of human MNCs in nude mice and suggest that the positive outcomes of nerve regeneration, angiogenesis, and reduction of inflammation may be attributed to the MNCs themselves.

The findings of electrophysiological examination revealed no statistically significant differences in the outcomes of NCV or stimulus threshold testing. However, the results of both testing parameters showed superior outcomes in both experimental groups compared to control (PBS group). Ultimately, establishing the enhanced efficacy of QQ-MNC cells will necessitate a prospective study involving real-life cases of peripheral nerve injury in human subjects.

### Limitations

This study also has some mentionable limitations. First, the 12-week duration of the study might exceed the optimal timeframe for effectively demonstrating the inflammatory responses and anti-inflammatory attributes of QQ-MNCs. The inflammatory reaction after peripheral nerve injury is important for initiating the nerve regeneration process – especially in Wallerian degeneration. Achieving a balance between pro-inflammatory and anti-inflammatory cytokines is the optimal condition for nerve repair. The next step for our team is to prove this concept both in the early stage of nerve repair, such as observing the results at 4 weeks after intervention, and in a chronic inflammatory context by developing a chronic inflammatory model [37]. The use of human stem cells in long-term experiments on mice (xenogeneic cells) can induce graft versus host-type immune responses. Therefore, the interpretation of results should be done cautiously due to this difference between species [45]. Second, for histological examination of nerve tissue, Luxol^®^ fast blue (LFB) stain may be more suitable than eosin relative to myelin sheath and fiber identification [46]. However, Luxol^®^ fast blue was not available at our center during this study, so we decided to use eosin stain instead. Third, for assessment of nerve regeneration, longitudinal section may be more suitable than the transverse section method. Our justification for selecting the transverse section method was because we wanted to observe nerve morphology, and to calculate and compare the integrated optical density (IOD) of the specimens in each group. To evaluate the nerve regeneration process, we divided the nerve specimen into proximal and distal nerve segments. In this study, we used the transverse section method to prove cellular engraftment by using anti-HNA to identify human stem cells in the nerve specimens of mice; however, the method we used may not be sufficient for confirming the cellular engraftment and migration process [4]. However, the magnification of the surgical microscope that we used to observe nerve morphology in this study may not have been sufficient to demonstrate the onion skin characteristic of degenerating fibers. Alternatively, we decided to evaluate and compare nerve morphology by measuring myelin thickness instead (the concept of measurement as in the reference [4]).

## Conclusions

QQ-MNCs exerted both direct and indirect positive effects on peripheral nerve repair. These cells were shown to enhance nerve repair by increasing axon fiber diameter, yielding more myelin thickness, and effectuating a higher percentage of nerve density. Immunohistochemistry staining confirmed the efficacy of nerve regeneration, the promotion of angiogenesis, and the lowering of inflammatory reactions. Moreover, the functional outcomes and wet-weight of the gastrocnemius muscle also demonstrated that QQ-MNCs help to maintain the function and morphology of the target organ following nerve injury. Taken together, the results of this study strongly suggest the potential use of QQ-MNCs for treating peripheral nerve damage.

## Supporting information

S1 Fig Cross-sectional study of gastrocnemius muscle specimens stained with hematoxylin and eosin (H&E) stain. (A) Phosphate buffered saline (PBS) group; (B) Peripheral blood mononuclear cells (PB-MNCs) group; and, (C) Quality and Quantity mononuclear cells (QQ-MNCs) group. (Bar = 50 μm).
(TIF)

S2 Fig Cross-sectional study of gastrocnemius muscle specimens stained with modified Gomori trichome (mGT) stain. (A) Phosphate buffered saline (PBS) group; (B) Peripheral blood mononuclear cells (PB-MNCs) group; and, (C) Quality and Quantity mononuclear cells (QQ-MNCs) group. (Bar = 50 μm).
(TIF)
