# Supplementary figures and images for: Efficacy of Quality and Quantity media-cultured mononuclear cells for promoting peripheral nerve regeneration in mouse model
Source: PLoS One. 2025 Apr 16;20(4):e0321457. doi: 10.1371/journal.pone.0321457 (PMC12002519; doi:10.1371/journal.pone.0321457)

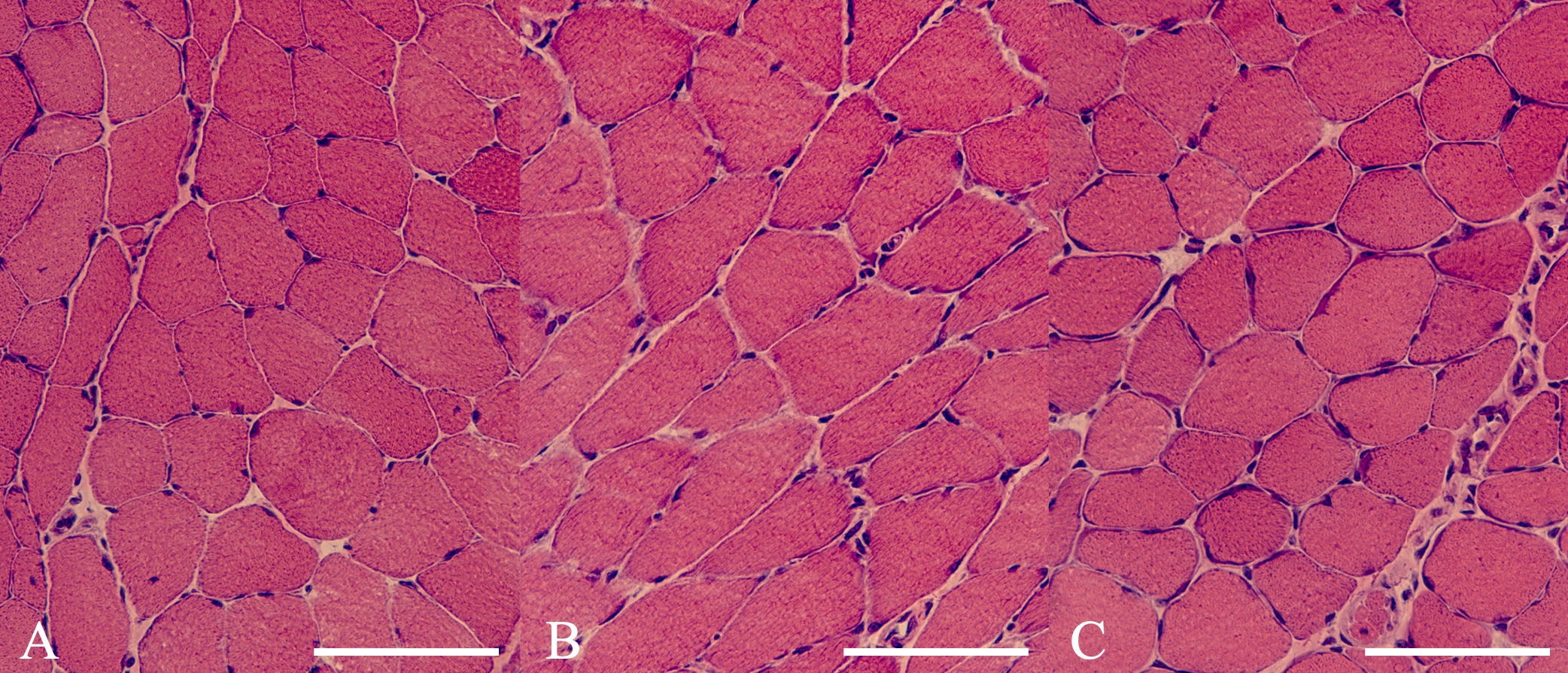

Supplement: S1 Fig — (A) Phosphate buffered saline (PBS) group; (B) Peripheral blood mononuclear cells (PB-MNCs) group; and, (C) Quality and Quantity mononuclear cells (QQ-MNCs) group. (Bar = 50 μm). (TIF) [file pone.0321457.s001.tif]

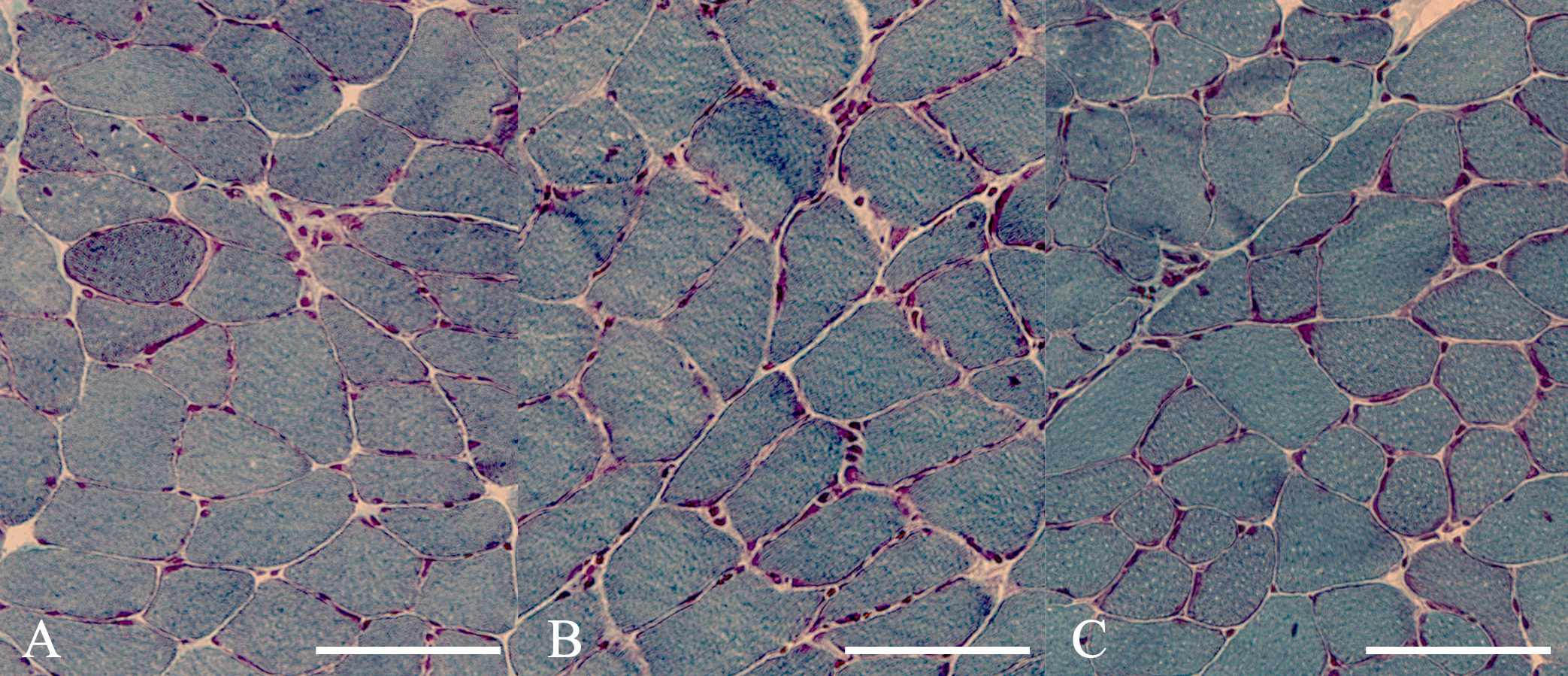

Supplement: S2 Fig — (A) Phosphate buffered saline (PBS) group; (B) Peripheral blood mononuclear cells (PB-MNCs) group; and, (C) Quality and Quantity mononuclear cells (QQ-MNCs) group. (Bar = 50 μm). (TIF) [file pone.0321457.s002.tif]
